# *In Vivo* Monitoring of the Antiangiogenic Effect of Neurotensin Receptor-Mediated Radiotherapy by Small-Animal Positron Emission Tomography: A Pilot Study

**DOI:** 10.3390/ph7040464

**Published:** 2014-04-16

**Authors:** Simone Maschauer, Tina Ruckdeschel, Philipp Tripal, Roland Haubner, Jürgen Einsiedel, Harald Hübner, Peter Gmeiner, Torsten Kuwert, Olaf Prante

**Affiliations:** 1 Department of Nuclear Medicine, Laboratory of Molecular Imaging and Radiochemistry, Friedrich Alexander University, Schwabachanlage 6, 91054 Erlangen, Germany; E-Mails: simone.maschauer@uk-erlangen.de (S.M.); tina_ruckdeschel@gmx.de (T.R.); philipp.tripal@uk-erlangen.de (P.T.); torsten.kuwert@uk-erlangen.de (T.K.); 2 Department of Nuclear Medicine, Innsbruck Medical University, Anichstr. 35, 6020 Innsbruck, Austria; E-Mail: roland.haubner@i-med.ac.at; 3 Department of Chemistry and Pharmacy, Medicinal Chemistry, Emil Fischer Center, Friedrich Alexander University, Schuhstraße 19, 91052 Erlangen, Germany; E-Mails: Jürgen.einsiedel@fau.de (J.E.); harald.huebner@fau.de (H.H.); peter.gmeiner@fau.de (P.G.)

**Keywords:** neurotensin receptor, positron emission tomography, radiotherapy, lutetium-177, RGD peptide, angiogenesis

## Abstract

The neurotensin receptor (NTS1) has emerged as an interesting target for molecular imaging and radiotherapy of NTS-positive tumors due to the overexpression in a range of tumors. The aim of this study was to develop a ^177^Lu-labeled NTS1 radioligand, its application for radiotherapy in a preclinical model and the imaging of therapy success by small-animal positron emission tomography (µPET) using [^68^Ga]DOTA-RGD as a specific tracer for imaging angiogenesis. The ^177^Lu-labeled peptide was subjected to studies on HT29-tumor-bearing nude mice *in vivo*, defining four groups of animals (single dose, two fractionated doses, four fractionated doses and sham-treated animals). Body weight and tumor diameters were determined three times per week. Up to day 28 after treatment, µPET studies were performed with [^68^Ga]DOTA-RGD. At days 7–10 after treatment with four fractionated doses of 11–14 MBq (each at days 0, 3, 6 and 10), the tumor growth was slightly decreased in comparison with untreated animals. Using a single high dose of 51 MBq, a significantly decreased tumor diameter of about 50% was observed with the beginning of treatment. Our preliminary PET imaging data suggested decreased tumor uptake values of [^68^Ga]DOTA-RGD in treated animals compared to controls at day 7 after treatment. This pilot study suggests that early PET imaging with [^68^Ga]DOTA-RGD in radiotherapy studies to monitor integrin expression could be a promising tool to predict therapy success *in vivo*. Further successive PET experiments are needed to confirm the significance and predictive value of RGD-PET for NTS-mediated radiotherapy.

## 1. Introduction

Neurotensin (NT) is a peptide consisting of 13 amino acids (pGlu-Leu-Tyr-Glu-Asn-Lys-Pro-Arg-Arg-Pro-Try-Ile-Leu) that has been originally isolated in 1973 by Carraway and Leeman from calf hypothalamus [1]. The biologically active sequence is the C-terminal part NT(8–13) (Arg-Arg-Pro-Tyr-Ile-Leu) [2] which binds to the three neurotensin receptor subtypes NTS1, NTS2, NTS3 [3] and has therefore been selected as a lead structure for medicinal chemists [4,5,6,7,8,9,10,11,12,13]. The NTS1 is upregulated in various tumor types including prostate, pancreas, mamma, lung and colon carcinoma [14,15,16]. In addition, NTS1 shows negligible expression in healthy tissues where these tumors arise from, making it a very specific molecular target for imaging and targeted cancer therapy [16]. NT(8–13) is rapidly degraded by endogenous peptidases *in vivo*, therefore a variety of studies addressed this issue by modifying the amino acid sequence of NT(8–13) [17,18,19,20,21,22,23].

Up to now, only a few NT(8–13) analogs have been radiolabeled with an isotope suitable for endoradiotherapy [24,25,26,27]. Among these rare examples, the ^188^Re-labeled peptide “NT-XIX” with the amino acid sequence (N^α^His)Ac-Arg-(*N*-CH_3_)-Arg-Pro-Dmt-Tle-Leu has been used for radiotherapy studies on tumor-bearing nude mice [24]. This radiopeptide showed promising effects, as the tumor growth in the animals was decreased at day 6 after begin of treatment. However, this study did not use successive diagnostic imaging experiments, such as positron emission tomography (PET) imaging, in a longitudinal setup for the prediction of therapy outcome.

**Figure 1:**
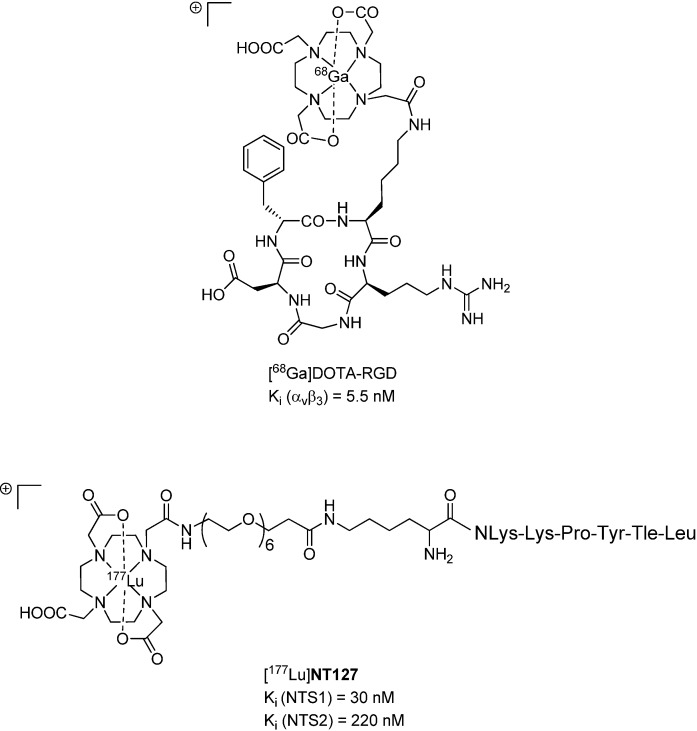
Chemical structures of [^68^Ga]DOTA-RGD [35] (**top**) and [^177^Lu]**NT127** (**bottom**).

Lutetium-177 is a β-emitter with a maximum energy of 0.5 MeV and a half-life of 6.7 days, displaying favorable physical properties compared to rhenium-188 (E_max_ = 2.1 MeV, t_½_ = 17 h). Lutetium-177 has a maximal tissue penetration of 2 mm, making it an optimal radionuclide for the irradiation of small tumors. Therefore, we aimed at developing a ^177^Lu-labeled NT-derivative, based on our previously reported metabolically stable amino acid sequence NLys-Lys-Pro-Tyr-Tle-Leu [28,29], and studied the applicability of this radiopeptide ([^177^Lu]**NT127**, Figure 1) for endoradiotherapy in NTS1-positive HT29-tumor-bearing nude mice.

The various integrin-specific PET tracers, all derived from pentacyclic RGD peptides [30,31], should be excellent molecular tools for monitoring early response of tumors to (radio)therapy, since antiangiogenic effects should occur at early stages after successful treatment [32,33,34]. Consequently, we studied the suitability of [^68^Ga]DOTA-RGD (Figure 1, [35]) for the noninvasive monitoring of tumor response to radiotherapy with [^177^Lu]**NT127** in a pilot study by successive PET imaging experiments, including days 7, 14, 21 and 28 after treatment and varying the administration of the dose from four fractionated doses (4 × 11–14 MBq, *n* = 4 mice) to a single high dose (1 × 50 MBq, *n* = 3 mice).

## 2. Experimental

### 2.1. General

Reagents, building blocks and dry solvents were obtained from commercial sources and were used as received. Analytical radio-HPLC was performed on an Agilent 1100 system (Agilent Technologies, Böblingen, Germany) equipped with a quaternary pump and variable wavelength detector and radio-HPLC detector D505TR (Canberra Packard, Schwadorf, Austria). Computer analysis of the HPLC data was performed using FLO-One software (Canberra Packard).

### 2.2. Synthesis of NT127

Unless otherwise noted, reactions were conducted without inert atmosphere. Microwave assisted (Discover^®^ microwave oven, CEM Corp., Kamp-Lintfort, Germany) peptide synthesis was carried out in glass tubes loosely sealed with a silicon septum. Remark: the development of overpressure was avoided by using DMF as the solvent. In between each irradiation step, intermittent cooling of the reaction mixture to a temperature of −10 °C was achieved by sufficient agitation in an ethanol-ice bath. Preparative RP-HPLC was performed using Agilent 1100 preparative series (column: Zorbax Eclipse XDB-C8, 21.2 × 150 mm, 5 µm particles [C8], flow rate. 10 mL /min, detection wavelength: 220 nm) and solvent systems as specified below. Purity and identity were assessed by analytical RP-HPLC (Agilent 1100 analytical series, column: Zorbax Eclipse XDB-C8 analytical column, 4.6 × 150 mm, 5 µm, flow rate: 0.5 mL/min, detection wavelength: 220 nm) coupled to a Bruker Esquire 2000 mass detector equipped with an ESI-trap. Solvent systems are specified below.

The peptide synthesis was achieved starting from commercially available Fmoc-Leu-Wang resin (Novabiochem^®^, Merck KGaA, Darmstadt, Germany, loading 0.64 mmol/g). Amino acids were incorporated as their commercially available derivatives in the following order: Fmoc-Tle-OH (a), Fmoc-Tyr(*t*Bu)-OH (b), Fmoc-Pro-OH (c), Fmoc-Lys(Boc)-OH (d), *N*-Fmoc-*N*-(4-Boc-aminobutyl)-Gly-OH (e, Aldrich, Taufkirchen, Germany), Boc-Lys(Fmoc)-OH (f), Fmoc-21-amino-4,7,10,13,16,19-hexaoxaheneicosanoic acid (g, Novabiochem) and 4,7,10-tris-*tert*-butoxycarbonylmethyl-1,4,7,10-tetraaza-cyclododec-1-yl)-acetic acid (DOTA-tri-*t*-Bu-ester, h, Chematech, Dijon, France). Elongation of the peptide chain was done by repetitive cycles of Fmoc deprotection applying 20% piperidine in DMF (microwave irradiation: 5× 5 s, 100 W), followed by 5 washings with DMF and subsequent peptide couplings using the following conditions: AA/PyBOP/diisopropylethylamine HOBt (5 eq/5 eq/5 eq/7.5 eq for a,b,c,d and 4 eq/4 eq/4 eq/6 eq for g) or AA/HATU/DIPEA (4 eq/4 eq/4 eq for e, 5 eq/5 eq/5 eq for f performing the coupling twice and 2.6 eq/2.6 eq/2.6 eq for h). The building blocks and reagents were dissolved in a minimum amount of DMF and the irradiation was performed 15× 10 s employing 50 W. After the last acylation step, the resin was 10× rinsed with CH_2_Cl_2_ and dried *in vacuo*. The on resin cleavage of *t-*Bu and Boc groups was achieved by stirring in a mixture of 10% v/v conc. H_2_SO_4_ in dioxane at 8 °C for 2 h and subsequent washing with 30% v/v DIPEA in CH_2_Cl_2_, followed by CH_2_Cl_2_ (2×). The cleavage from the resin was performed using a mixture of TFA/phenol/H_2_O/triisopropylsilane 88:5:5:2 for 2 h. After evaporation of the solvent and precipitation in *tert*-butylmethyl ether, the crude peptide was purified using preparative RP-HPLC (eluent: CH_3_CN (A) + 0.1% HCO_2_H in H_2_O (B) applying a linear gradient 3%–20% A in 97%–80% B in 16 min, t_R_: 13.9 min) and subsequent lyophilization to afford the peptide as the formate salt. Peptide purity and identity was assessed by analytical HPLC. System 1:10%–40% CH_3_OH in 90%–60% H_2_O + 0.1% HCO_2_H in 18 min, purity: 92% (t_R_: 17.3 min); system 2:3%–30% CH_3_CN in 97%–70% H_2_O + 0.1% HCO_2_H in 26 min, purity: 93% (t_R_: 17.4 min). [M+H]^+^; calcd for C_75_H_132_N_15_O_23_: 1611.0, found: 1611.0.

### 2.3. Synthesis of [^175^Lu]NT127

**NT127** (3.6 mg, 2 µmol) was dissolved in HEPES (0.5 M, 600 µL, pH 5) and Lu(NO_3_)_3_ (1.5 mg, 4 µmol) was added. After 10 min at 98 °C [^175^Lu]**NT127** was purified by semipreparative HPLC (Kromasil C8, 125 × 8 mm, 10%–50% acetonitrile (0.1% TFA) in water (0.1% TFA) in a linear gradient over 25 min, 4 mL/min, t_R_ = 9.8 min). Peptide purity and identity was assessed by analytical HPLC. System 1: 10%–40% CH_3_OH + 0.1% HCO_2_H in 90%–60% H_2_O in 18 min, Purity: >99% (t_R_: 16.8 min). [M]^+^; calcd for C_75_H_129_N_15_O_23_Lu: 1782.9, found: 1782.8.

### 2.4. Neurotensin Receptor Binding Experiments

Receptor binding data were determined according to protocols as described previously [6,36]. In detail, NTS1 binding was measured using homogenates of membranes from CHO cells stably expressing human NTS1 at a final concentration of 2 μg/well and the radioligand [^3^H]neurotensin (specific activity 116 Ci/mmol; PerkinElmer, Rodgau, Germany) at a concentration of 0.50 nM. Specific binding of the radioligand was determined at a K_D_ value of 0.69 nM and a B_max_ of 6200 fmol/mg protein. Nonspecific binding was determined in the presence of 10 μM neurotensin. NTS2 binding was done using homogenates of membranes from transiently transfected HEK 293 cells at a concentration of 20 μg/well together with 0.50 nM of [^3^H]NT(8–13) (specific activity 136 Ci/mmol; custom synthesis by GE Healthcare, Freiburg, Germany) at a K_D_ value of 1.3 nM and a B_max_ of 800 fmol/mg protein. Nonspecific binding was determined in the presence of 10 μM NT(8–13). Protein concentration was generally determined by the method of Lowry using bovine serum albumin as standard [37]. Data analysis of the competition curves from the radioligand binding experiments was accomplished by non-linear regression analysis using the algorithms in PRISM5.0 (GraphPad Software, San Diego, CA, USA). EC_50_ values derived from the resulting dose response curves were transformed into the corresponding K_i_ values according to the equation of Cheng and Prusoff [38].

### 2.5. Synthesis of [^177^Lu]NT127

**NT127** (4 nmol) was dissolved in 5 µL of metal-free water and HEPES (0.5 M, 200 µL, pH 5) and n.c.a. [^177^Lu]LuCl_3_ (20–50 µL, 50–100 MBq, Isotope Technologies Garching (ITG) GmbH, Garching, Germany) was added. After 10 min at 98 °C the reaction mixture was diluted with isotonic saline (0.9%) and [^177^Lu]**NT127** was used without further purification for *in vitro* and *in vivo* experiments. The radiochemical yield and radiochemical purity was determined from an aliquot taken from the reaction mixture by radio-HPLC (t_R_ = 2.20 min; Chromolith RP-18e, 10 × 4.6 mm, 10%–50% acetonitrile (0.1% TFA) in water (0.1% TFA) in a linear gradient over 5 min, 4 mL/min) and was determined >98%.

### 2.6. Synthesis of [^68^Ga]DOTA-RGD

To the DOTA-conjugated precursor c(RGDfK(DOTA)) [35] (10 nmol) in acetate buffer (2.5 M, 140 µL) was added an aliquot of [^68^Ga]GaCl_3_ (200 MBq in 500 µL, 0.6 M HCl), freshly eluted from a ^68^Ge/^68^Ga generator (IDB Holland BV (Baarle-Nassau, The Netherlands)/iThemba LABS (Cape Town, South Africa)), resulting in a final pH of 4.0. After incubation for 10 min at 98°C, the solution was neutralized by the addition of sodium bicarbonate (1 M, 120 µL). The radiochemical yield was >98% as determined by radio-HPLC (t_R_ = 1.30 min; Chromolith RP-18e, 10 × 4.6 mm, 10%–50% acetonitrile (0.1% TFA) in water (0.1% TFA) in a linear gradient over 5 min, 4 mL/min) and the specific activity was determined to be 10–15 GBq/µmol. The resulting solution was used for small animal PET studies without further purification.

### 2.7. Stability of [^177^Lu]NT127

An aliquot of [^177^Lu]**NT127** (30 µL, about 2 MBq) was added to human serum (200 µL) and incubated at 37 °C. Aliquots (25 µL) were taken at various time intervals (5–210 min) and quenched in ethanol/water (1:1, 100 µL). The samples were centrifuged, and the supernatants were analyzed by radio-HPLC (t_R_ = 2.20 min; Chromolith RP-18e, 10 × 4.6 mm, 10%–50% acetonitrile (0.1% TFA) in water (0.1% TFA) in a linear gradient over 5 min, 4 mL/min).

### 2.8. Cell Culture

The human NTS1-expressing cell line HT29 (ECACC NO 91072201) [39], was grown in culture medium (McCoy’s 5a medium containing glutamine (2 mM) supplemented with fetal bovine serum (FBS, 10%)) at 37 °C in a humidified atmosphere of 5% CO_2_. Cells were routinely subcultured every 3–4 days. Viability of the cells was determined by staining with trypan blue and was >90% for all cells used in *in vitro* and *in vivo* studies.

### 2.9. Internalization and Efflux Studies with [^177^Lu]NT127

Three days before experimental use, approximately 250,000 HT29 cells were seeded in 24-multiwell plates. The medium was changed to binding buffer (culture medium supplemented with 1% bovine serum albumin (BSA), HEPES (10 mM), chymostatin (2 mg/L) and soybean trypsin inhibitor (100 mg/L), 0.5 mL) and [^177^Lu]**NT127** (0.3 MBq, 10 µL) was added to each well. Cells were incubated for different time intervals (5, 15, 30, 60, 90 and 120 min) at 37 °C, before the incubation was terminated by placing the cells on ice, aspirating the incubation buffer and washing the cell layer twice with ice-cold PBS. The cell layers were washed twice with an acidic washing buffer (20 mM sodium acetate, pH 5) to remove non-sequestered radioactivity. Cells were harvested with NaOH (0.1 M, 1 mL) and counted in a gamma counter (Wizard Wallac, Perkin Elmer). Nonspecific binding was determined in the presence of neurotensin (1 µM). Two independent experiments were performed in quadruplicate.

For efflux studies HT29 cells were seeded in 24-multiwell plates and incubated with [^177^Lu]**NT127** as described above. After an incubation time of 30 min at 37 °C the plates were placed on ice and the cells were washed with ice-cold PBS (500 µL), acidic wash buffer (500 µL, 1 min) and again with ice-cold PBS (500 µL). Subsequently, fresh binding buffer (500 µL) was added to each well and the plates were incubated at 37 °C. After 5 min, 15 min, 30 min, 45 min, 60 min and 90 min the plates were placed on ice, the supernatant was transferred to a counting tube, the cells were washed once with ice-cold PBS (500 µL) and this washing PBS was combined with the respective supernatant in the counting tubes. Cells were harvested with NaOH (0.1 M, 1 mL) and cell suspensions and washing solutions were counted in a gamma counter (Wizard Wallac). The experiment was performed twice in quadruplicate.

### 2.10. Animal Model (Nude Mice Bearing HT29 Xenografts)

All animal experiments were performed in compliance with the protocols approved by the local animal protection authorities (Regierung Mittelfranken, Ansbach, Germany, No. 54-2532.1-22/10). Female athymic nude mice (nu/nu) were obtained from Harlan Winkelmann GmbH (Borchen, Germany) at 4 weeks of age and were kept under standard conditions (12 h light/dark) with food and water available ad libitum. HT29 cells were harvested and suspended in sterile PBS at a concentration of 10^7^ cells/mL. Each mouse was injected subcutaneously in the lower region of the left shoulder on the back with viable cells (2 × 10^6^) in PBS (200 μL).

### 2.11. Biodistribution Studies

10–14 days after inoculation of the HT29 cells (tumor weight: 200–700 mg), the mice (about 10–12 weeks old with about 35–40 g body weight) were used for biodistribution studies. HT29 xenografted mice were injected with [^177^Lu]**NT127** (1–3 MBq/mouse) intravenously into the tail vein. They were sacrificed by cervical dislocation 1 h, 4 h and 24 h after injection. Tumors and other tissues (blood, lung, liver, kidneys, heart, spleen, brain, muscle, femur and intestine) were rapidly removed and weighed. Radioactivity of the dissected tissues was measured using a gamma counter (Wallac Wizard, Perkin Elmer). Results were expressed as percentage of injected dose per gram of tissue (% ID/g), and tumor-to-organ ratios were calculated. Two randomly chosen mice were used for the determination of nonspecific tracer uptake *in vivo*. These blocking experiments were carried out by intravenous coinjection of mice with [^177^Lu]**NT127** together with NT4 [28] (100 μg/animal). These mice were sacrificed by cervical dislocation at 4 h post injection (p.i.) and organs and tissue were removed, weighed and counted as described above.

### 2.12. Radiotherapy Studies with [^177^Lu]NT127 and Small Animal PET Imaging

5–6 days after inoculation of the HT29 cells (tumor diameter: 4–5 mm), the mice (about 10–12 weeks old with about 35–40 g body weight) were divided into four groups. The control group (*n* = 8) received i.v. the vehicle (0.5 nmol **NT127** in 100 µL saline) with no activity at the same days as the treated animals. Treated group 1 (*n* = 4) received i.v. 28–38 MBq/mouse [^177^Lu]**NT127** in two fractionated doses of 11–15 MBq (at days 0) and 17–23 MBq at day 6, treated group 2 (*n* = 3) received a single dose of 49–51 MBq/mouse [^177^Lu]**NT127** (at day 0) and treated group 3 (*n* = 4) received 50–51 MBq/mouse [^177^Lu]**NT127** in four fractionated doses of 11–14 MBq/mouse (at days 0, 3, 6 and 10). Each dose contained between 0.3–0.7 nmol **NT127**. The body weight of the mice was determined three times a week and the diameter of the tumors was measured with a calliper three times a week for 30–33 days starting the day of the first injection (day 0). An additional group of three animals was injected with a single low dose of 21 MBq/mouse. These were also used for histological staining of tissue samples from kidney and liver.

PET scans and image analysis were performed using a microPET rodent model scanner (Inveon, Siemens Healthcare, Erlangen, Germany). About 3–8 MBq of [^68^Ga]DOTA-RGD was intravenously injected into each mouse at day 7, 14, 21 and 28 after the first [^177^Lu]**NT127**-treatment under isoflurane anesthesia (4%). Animals were subjected to a 15 min scan starting from 45 min p.i. After 3D-OSEM iterative image reconstruction with decay and attenuation correction, regions of interest (ROIs) were drawn over the tumor region, and the mean within a tumor was converted to standard uptake values (SUV) considering the injected activity and body weight.

### 2.13. Histology of Tissue Samples from Kidney and Liver

28 days after first treatment the mice were sacrificed by cervical dislocation and kidneys and liver were dissected and immediately fixed in 4% neutral buffered formalin at room temperature overnight and then stored in phosphate buffered saline at 4 °C. For histological examinations the tissue was paraffin-embedded, cut in four-micrometer sections and stained with Masson-Goldner trichrome. Quantification of tissue inflammation was done using a digital light microscope (Axioplan 2 imaging, Zeiss) with the software Metafer 4 (v. 3.9.1) and VSlide (v. 1.0.125). A virtual grid was placed over the sections and the quantitative detection of inflammation per visual field was carried out according to the following scheme: none (0), low (1), moderate (2), much (3).

## 3. Results and Discussion

**Scheme 1:**
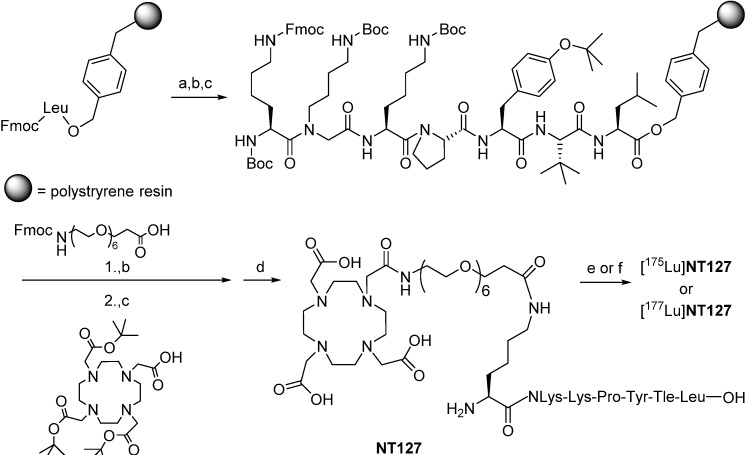
(Radio)synthesis of [^175^Lu]**NT127** and [^177^Lu]**NT127**.

Our plan of synthesis was based on our previous publications on metabolically stabilized NT(8–13) derivatives (Scheme 1) [7,28,29].

*Reagents and conditions*: (**a**) Fmoc-deprotection: piperidine/DMF (1:4), μ~: 5× 5 s, 100 W, 5 × cooling to −10 °C; (**b**) peptide coupling for Fmoc-Tle-OH, Fmoc-Tyr(*t*Bu)-OH, Fmoc-Pro-OH, Fmoc-Lys(Boc)-OH, Fmoc-21-amino-4,7,10,13,16,19-hexaoxaheneicosanoic acid: Fmoc-AA-OH, PyBOP, DIPEA, HOBt, DMF, μ~, 15× 10 s, 50 W, 15× cooling to −10 °C; **(c)** for *N*-Fmoc-*N*-(4-Boc-aminobutyl)-Gly-OH, Boc-Lys(Fmoc)-OH, DOTA-tri-*t*-Bu-ester: Fmoc-AA-OH, HATU, DIPEA, DMF, μ∼-assisted coupling (see **b**); (**d**) deprotection/cleavage of the peptide: (1) H_2_SO_4_/dioxane (1:9), 8 °C, 2 h; (2) DIPEA/CH_2_Cl_2_ (3:7); (3) TFA, phenol, H_2_O, triisopropylsilane 88:5:5:2, 2 h, followed by RP-HPLC; (**e**) Lu(NO_3_)_3_, 0.5 M HEPES pH 5, 98 °C, 10 min or **(f)**n.c.a. [^177^Lu]LuCl_3_, 0.5 M HEPES pH 5, 98 °C, 10 min.

In detail, we applied solid-phase methods with repetitive cycles of Fmoc deprotection with piperidine and acylation with the respective Fmoc-protected amino acids. Amino acid activation was done in the presence of PyBOP/HOBt, thus allowing us to incorporate *t*-leucine, tyrosine, proline, lysine, and amino-4,7,10,13,16,19-hexaoxaheneicosanoic acid. The more powerful coupling agent HATU was employed for the attachment of *N*-(4-aminobutyl)-glycine, for the lysine linker as well as for the chelator 1,4,7,10-tetraazacyclododecane-1,4,7,10-tetraacetic acid (DOTA), which was coupled as the commercially available tri-*t*-butyl ester derivative. Microwave acceleration proved to be advantageous for both Fmoc deprotection and acylation reactions. For a safe peptide cleavage without concomitant *t*-butylation of the peptide side chains, we decided to apply our recently published two step protocol removing the *t*-butyl-based protective groups by a pre-treatment with 10% sulfuric acid in dioxane at 8 °C at first [10]. Thereafter, final cleavage from the resin with TFA resulted in the formation of crude peptide with acceptable amounts of *t*-butylated byproduct, sufficiently pure to allow successful high-performance liquid chromatography (HPLC) purification. The synthesis of the lutetium complex [^175^Lu]**127** was accomplished by stirring **NT127** for 10 min with an excess of Lu(NO_3_)_3_ (2 equiv) in 0.5 M HEPES buffer (pH 5) at 98 °C, followed by RP-HPLC purification (Scheme 1). Electrospray ionization-mass spectrometry revealed the [M]^+^ peak of [^175^Lu]**127**, confirming previous studies on DOTA-conjugated peptides with one free deprotonated carboxylate group at physiological pH that does not participate with the pseudo-octahedral geometry of the Ga-DOTA complex of the peptide [40].

After verification of the purity of **NT127** (≥92%) and of the reference compound [^175^Lu]**NT127** (>99%), the lutetium complex [^175^Lu]**NT127** was subjected to receptor binding studies for the neurotensin receptor subtypes NTS1 and NTS2. The results revealed substantial NTS1 subtype selectivity with a K_i_ value for NTS1 of 30 nM (Table 1).

**Table 1:** Receptor binding data of [^175^Lu]**NT127** in comparison with the reference ligands neurotensin and NT(8–13) employing human NTS1 and NTS2 ^a^.

Compound	K_i_ value ± SEM [nM]	
	K_i_ [nM] NTS1 ^b^ [^3^H]neurotensin	K_i_ [nM] NTS2 ^c^ [^3^H]NT(8–13)
Neurotensin	0.51 ± 0.06 ^d^	4.9 ± 0.3
NT(8–13)	0.29 ± 0.03	1.4 ± 0.1 ^d^
[^175^Lu] **NT127**	30 ± 6	220 ± 93

^a^ Ki values ± SEM are the means of three individual experiments each done in triplicate; ^b^ Membranes from CHO cells stably expressing human NTS1; ^c^ Homogenates from HEK cells transiently expressing human NTS2; ^d^ K_d_ values ± SEM are the means of 13–20 individual saturation experiments each done in quadruplicates.

**Figure S1:**
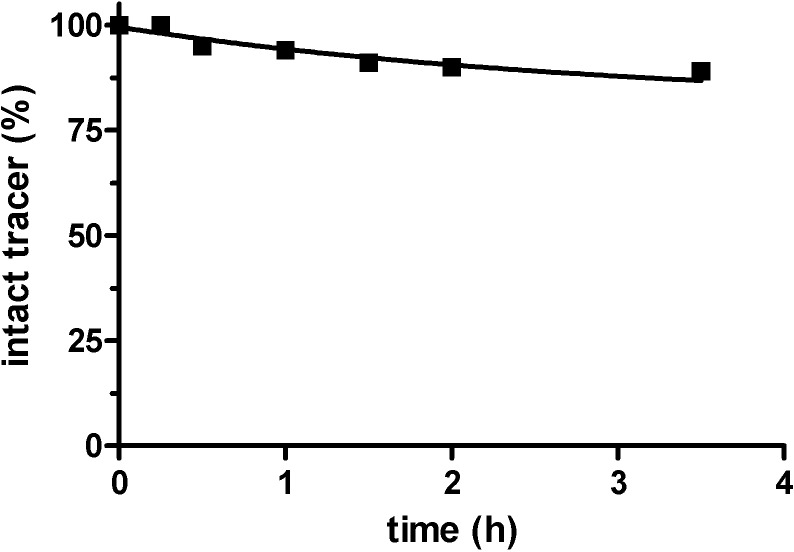
Radiochemical purity of [^177^Lu]**NT127** after incubation in human serum in vitro at 37 °C for up to 3.5 h as determined by radio-HPLC.

**Figure S2:**
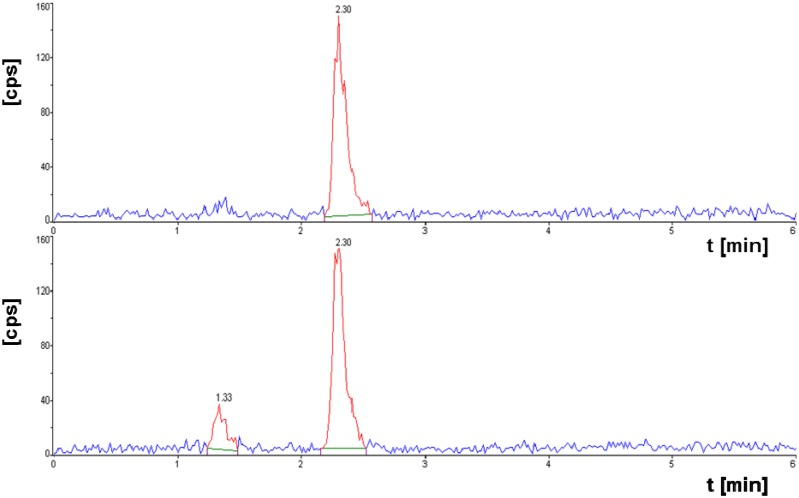
[^177^Lu]**NT127** after incubation in human serum in vitro at 37 °C for 15 min (**upper HPLC chromatogram**), and for 3.5 h (**lower HPLC chromatogram**).

Starting from **NT127**, the radiosynthesis of [^177^Lu]**NT127** was performed in HEPES buffer at 98 °C to give [^177^Lu]**NT127** in high radiochemical yield and high radiochemical purity (> 98%) without the need for further purification (Scheme 1). The stability of the tracer in human serum *in vitro* was 89% after incubation at 37 °C for 3.5 h (Figure S1 and Figure S2).

**Figure 2:**
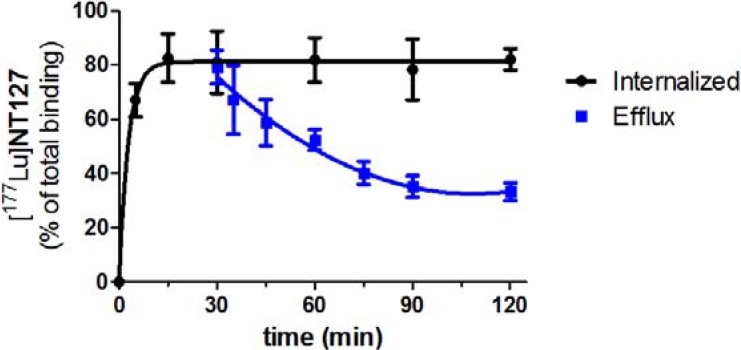
Internalization and efflux of [^177^Lu]**NT127** in human HT29 cells. Each data point represents the mean ± standard deviation of two experiments performed in quadruplicate.

The amount of internalization [^177^Lu]**NT127** was determined *in vitro* using HT29 cells. [^177^Lu]**NT127** revealed an internalization rate of 82% ± 9% after 15 min which remained constant over 120 min (Figure 2). To determine the efflux from the cells, [^177^Lu]**NT127** was allowed to internalize for 30 min into HT29 cells, then the cells were washed with acid buffer to remove surface bound radioactivity, and fresh media was added. After 90 min there was still a high amount of internalized [^177^Lu]**NT127** of 42% ± 5% (Figure 2).

**Figure 3:**
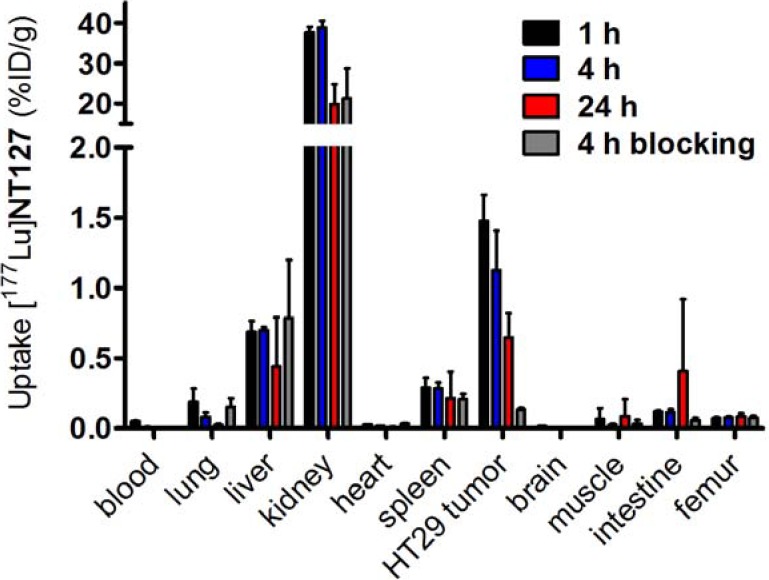
Biodistribution of [^177^Lu]**NT127** in HT29 xenografted nude mice 1 h , 4 h and 24 h after injection and 4 h after injection of [^177^Lu]**NT127** together with **NT4** (100 µg/mouse, “blocking”). Each data point represents the mean ± standard deviation of tissue samples from two mice.

The biodistribution studies of [^177^Lu]**NT127** were carried out with HT29 tumor-bearing nude mice and showed a strong accumulation of the tracer in the kidney with 38 ± 1% ID/g at 1 h post-injection (p.i.), which was reduced to 20% ± 5% ID/g at 24 h p.i. (Figure 3). [^177^Lu]**NT127** showed the second highest accumulation in the HT29 tumor at 1 h p.i. (1.5% ± 0.2% ID/g), whereas after 24 h the tracer was partially washed out again (0.6% ID/g). In the liver the radioactivity concentration was 0.7% ± 0.1% ID/g after 1 h and 0.4% ID/g after 24 h. The clearance from blood was very fast, resulting in 0.05% ID/g in blood at 1 h p.i. which was reduced to 0.01% ID/g at 24 h p.i., providing an excellently high tumor-to-blood ratio of 30 (60 min p.i.) and 60 (24 h p.i.), respectively.

In order to determine the specific binding of [^177^Lu]**NT127** *in vivo*, two HT29 tumor-bearing nude mice were co-injected with [^177^Lu]**NT127** and the high-affinity NTS1 ligand **NT4**[28]. In the HT29 tumor the accumulation of [^177^Lu]**NT127** was significantly reduced at 4 h p.i. by the co-injection of **NT4** from 1.1% ID/g to 0.1% ID/g, indicating the specificity of tracer uptake in the HT29 tumors.

**Figure 4:**
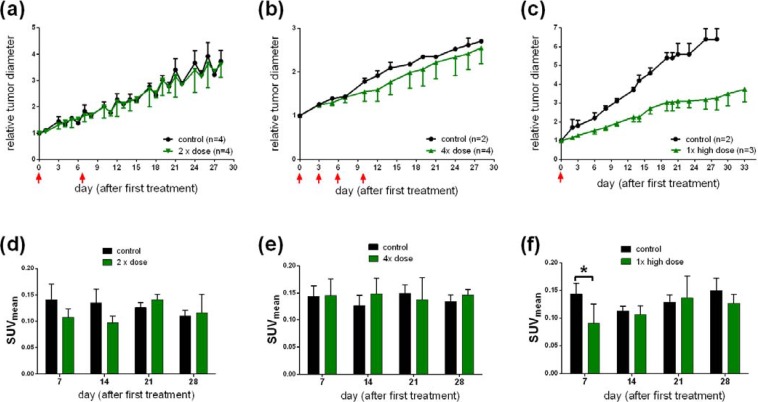
**(a, b, c)** Change in tumor growth after a single high **(c)** dose or a two-time **(a)** or four-time **(b)** sequential dose of [^177^Lu]**NT127**. **(d, e, f)** Standard uptake values (SUVmean) of [^68^Ga]DOTA-RGD in HT29 tumor-bearing nude mice at day 7, 14, 21 and 28 after injection of a single **(f)**; two-time **(d)**; or four-time **(e)** dose of [^177^Lu]**NT127**. *p < 0.05, *n* = 8 (sham-treated animals) *vs.* *n* = 3 (treated animals).

**Figure S3:**
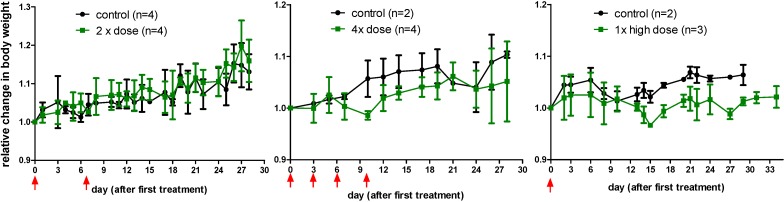
Relative change in body weight of the nude mice after a single high (**right**) dose or a two-time (**left**) or four-time (**middle**) sequential dose of [^177^Lu]**NT127** in comparison with sham-treated animals (control). Data are expressed as mean ± standard deviation.

To study the effect of [^177^Lu]**NT127** as a radiotherapeutical agent, HT29 tumor-bearing nude mice were injected with different doses of [^177^Lu]**NT127** and the tumor growth as well as the body weight was measured every third day over a 30 day-period and compared with mice injected with vehicle only. In all experiments, the course of the body weight of the treated animals during therapy did not reveal a static decrease, indicating the physical fitness of the animals (Figure S3). However, a statistical analysis of the differences in body weight has not been performed due to the low number of animals used in this initial study. In group 1 that received two doses of 11–15 MBq and 17–23 MBq for each animal at day 0 and day 6, respectively, no change in tumor growth was observed when compared to the control group (Figure 4a). The animals in treatment group 2, receiving four fractionated doses of 11–14 MBq/mouse at days 0, 3, 6 and 10, also did not show any significant change in the mean tumor growth (Figure 4b). It is worth mentioning that two of the four treated mice of this group showed reduced tumor growth, however, the mean value over all four animals did not reveal any significant difference in the relative tumor growth. In contrast to the fractionated administration, the animals in treatment group 3 received a single high dose of 50 MBq per animal at the beginning of radiotherapy. These animals showed a significant reduction of about 50% in the mean tumor growth for all treated animals compared to the sham-treated controls (Figure 4c).

**Figure 5:**
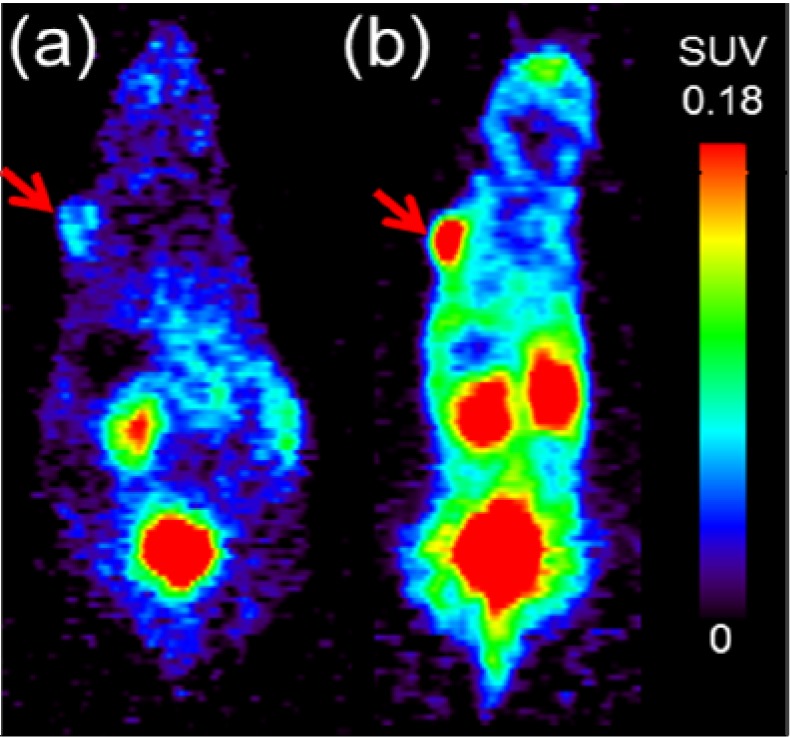
Representative PET scans at 45–60 min p.i. of [^68^Ga]DOTA-RGD obtained from HT29 tumor-bearing nude mice **(a)** at day 7 after radiotherapy with [^177^Lu]**NT127** (1× high dose, 50 MBq) *vs.* **(b)** sham-treated at day 7 after begin of treatment. Both images are normalized to the same standard uptake value (SUV_mean_) scale. Red arrows indicate the HT29 tumor.

Next, we investigated the use of [^68^Ga]DOTA-RGD to monitor the response of tumors to the radiotherapy with [^177^Lu]**NT127** in the four nude mice groups. Therefore, PET scans with [^68^Ga]DOTA-RGD were performed at day 7, 14 21 and 28 after first treatment. As shown in Figure 4d–f the only significant change in tumor uptake values (SUV_mean_) can be observed at day 7 after treatment by the single high dose of 50 MBq (Figure 4f, *p* < 0.05, *n* = 8 (sham-treated animals) *vs.n* = 3 (treated animals)). The decreased uptake of the RGD tracer indicated a lower degree of α_v_β_3_ integrin expression in the tumor region at day 7 after treatment. The difference between control and treated animals was also confirmed by the µPET image analysis of a representative pair of animals, when the treated animal indicated a significantly lower uptake of [^68^Ga]DOTA-RGD in the HT29 tumor region compared to the corresponding sham-treated animal, as shown in Figure 5. However, we could not detect any significant changes in the tumor uptake of [^68^Ga]DOTA-RGD between treated and untreated animal groups at later time points of more than seven days after treatment (Figure 4f). This result might reflect the response of HT29 tumors toward radiation, resulting in regeneration of maximal integrin expression in the tumor region that did not differ significantly any more from the level that was found in sham-treated animals at day 7 and later after treatment. Noteworthy, HT29 are well-known to show a radiation-induced increase in radioresistance at low doses [41], so this phenomenon could account for the weak response of HT29 tumors toward fractionated therapy by administration of four equal doses (group 3, Figure 4b).

Taken together, the single high-dose endoradiotherapy with 50 MBq [^177^Lu]**NT127** showed a substantial mean effect of 50% reduced tumor diameter, which is comparable to the result reported by Schubiger and coworkers for the rhenium-labeled peptide analog [24]. Moreover, preliminary µPET experiments identified day 7 after treatment as a potential time point for the analysis of integrin expression by RGD-PET, trying to identify an anti-angiogenic effect as an early predictor of response to endoradiotherapy with [^177^Lu]**NT127**.

**Figure 6:**
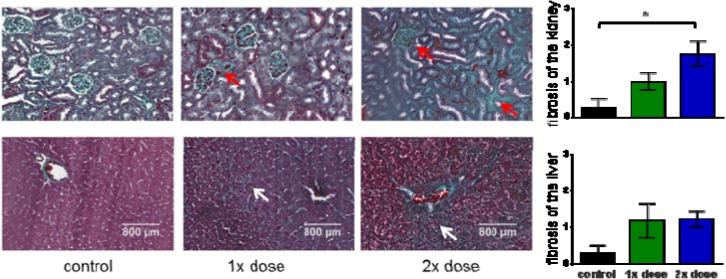
Histological examinations of kidney (**upper row**) and liver (**lower row**). Masson-goldner trichrome staining of liver and kidney sections from mice which received a single low dose of 21 MBq (1× dose) or two fractionated doses of 13 MBq and 19 MBq (2× dose) in comparison with sham-treated animals (control). Red arrows indicate fibrotic lesions in kidney. White arrows indicated fibrotic lesions in liver. All images are representative photographs captured at a magnification of ×10 (the scale bar represents 800 µm). Bar charts: The number of fibrotic tissue damages was quantified for whole sections of each tissue at comparable position and evaluated according to the following classification: 0/no fibrosis, 1/few fibrosis, 2/medium fibrosis, 3/many fibrosis. The asterisk indicated a significant difference (*p* < 0.05, one-way Anova test).

The clearance of [^177^Lu]**NT127** predominantly occurred through the kidney and to a lower portion through the liver (Figure 3), so that these organs received the highest radiation dose during tumor therapy. Therefore, we performed histological staining to study potential tissue damage, e.g., fibrotic lesions, in kidney and liver. In particular, both the glomeruli and the tubules in the kidney sections from animals that received a two-time consecutive dose show strong fibrosis compared to sham-treated control animals (Figure 6). In the corresponding liver sections, both the singular and the sequential dose led to increased tissue damage (Figure 6). This finding might hamper the translation of the NTS1-mediated radiotherapy approach with [^177^Lu]**NT127** into human application in the future.

In medical oncology, it is desirable to predict as early as possible whether an individual tumor responds to treatment. Structural imaging techniques such as X-ray computerized tomography (CT) or magnetic resonance imaging (MRI) capitalize on changes in tumor size for this purpose. However, a treatment-induced decrease of tumor size does often not occur until weeks to months after institution of treatment; furthermore, e.g., in lymphomas, non-neoplastic scar tissue may persist despite eradication of the tumor cells, leading to false-negative CT or MRI results. Therefore, the possible value of the molecular imaging modality PET for this purpose has been subject of considerable research efforts in the last decade (for a review, see [42]). The PET measurement of glucose consumption using [^18^F]fluorodeoxyglucose has, indeed, been proven to be of some value in this regard and, in particular, for establishing proof of treatment response in lymphomas, colorectal carcinomas, and gastrointestinal stroma tumors.

Changes in tumor vasculature are thought to have an important role in determining tumor response to radiotherapy [43]. Furthermore, radiation treatment does not only destroy the neoplastic cells, but also the vascular bed of the tumor as endothelial cells are even more sensitive to radiation than most cancer cells. Therefore, in this study, in addition to tumor volume, we analysed integrin expression to predict treatment effect. In the single-dose group that responded by significant tumor shrinkage to therapy radioreceptor treatment produced also an early decrease in RGD tracer uptake as measured by PET. However, this was only short-lived, although the treated animals consistently had lower tumor volumes than those in the control group until the end-point of treatment at day 28. One possible explanation for this phenomenon is provided by inflammatory changes occurring after initial radiation damage to the vascular bed of the tumors and to the tumor cells themselves. Indeed, some evidence for that could be seen in our histopathological specimens. To date, literature is scarce with regard to alterations of integrin tissue expression caused by radiation treatment. Therefore, this interpretation of our results must remain speculative, awaiting confirmation by future research.

## 4. Conclusions

With [^177^Lu]**NT127** in our hands, we offer an agonist radiopeptide with high-affinity to NTS1, providing the option to study endoradiotherapy with that radiopeptide in preclinical animal models. In NTS1-positive HT29-tumor-bearing nude mice, we observed decreased tumor progression after administration of a single high-dose of the radiopeptide. In the sequentially treated animals, the decreased tumor progression was less pronounced visible when analyzing the tumor diameter. However, our preliminary PET imaging data at day 7 after treatment with a single high dose of [^177^Lu]**NT127** suggested decreased uptake values of [^68^Ga]DOTA-RGD compared to sham-treated controls. Further longitudinal PET imaging studies are needed to confirm the statistical significance and predictive value of RGD-PET for NTS1-mediated radiotherapy.
